# Supplementary material for: The Bulimulidae (Mollusca: Pulmonata) from the Región de Atacama, northern Chile
Source: PeerJ. 2015 Nov 5;3:e1383. doi: 10.7717/peerj.1383 (PMC4647600; doi:10.7717/peerj.1383)
Supplement: Supplemental Information 1 [file peerj-03-1383-s001.docx]

**RESUMEN**

El género bulimúlido *Bostryx* Troschel, 1847 es el género de caracoles terrestres más rico en especies presente in Chile, con la mayoría de sus especies encontradas solo en el norte del país, usualmente en zonas áridas costeras. Este género ha sido poco estudiado en Chile y hay poca información en su distribución, diversidad o ecología. Aquí, por primera vez, se reporta un análisis formal de la diversidad de bulimúlidos en la Región de Atacama, norte de Chile. De las diecisiete especies registradas para el área, la mayoría de ellas fueron efectivamente encontradas en las colecciones de campo y una especie fue registrada solo en la literatura, cinco especies son descritas por primera vez: *Bostryx* *ancavilorum* sp. nov., *Bostryx breurei* sp. nov., *Bostryx* *calderaensis* sp. nov., *Bostryx ireneae* sp. nov. y *Bostryx valdovinosi* sp. nov. y ademas, se extienden las distribuciones geográficas de siete especies. Los resultados revelan que la Región de Atacama es la región mas rica en caracoles terrestres después del Archipielago de Juan Fernández. Todas las especies de moluscos terrestres que ocurren en el área son endemicas de Chile, la mayoría de ellas poseen distribuciones geográficas restringidas a lo largo de las zonas costeras del país, ninguna de ellas se encuentra protegida por la ley. Futuros estudios de campo en el norte de chile probablemente revelen nuevos registros o nuevas especies especies de caracoles terrestres.
